# Dysregulated Signaling Pathways in Canine Mammary Tumor and Human Triple Negative Breast Cancer: Advances and Potential Therapeutic Targets

**DOI:** 10.3390/ijms26010145

**Published:** 2024-12-27

**Authors:** Chen Mei, Ying Liu, Zhenyi Liu, Yan Zhi, Zhaoling Jiang, Xueze Lyu, Hongjun Wang

**Affiliations:** 1 Institute of Animal Husbandry and Veterinary Medicine, Beijing Academy of Agriculture and Forestry Sciences, Beijing 100097, China; meichen@baafs.net.cn (C.M.); liuying@baafs.net.cn (Y.L.); liuzylynn@163.com (Z.L.); bjzhiyan@sina.com (Y.Z.); m13483551153@163.com (Z.J.); 2 College of Veterinary Medicine, China Agricultural University, Beijing 100193, China

**Keywords:** human breast cancer, canine mammary tumor, signaling pathways, drug targets

## Abstract

In 2022, human breast cancer (HBC) and canine mammary tumors (CMTs) remained the most prevalent malignant tumors worldwide, with high recurrence and lethality rates, posing a significant threat to human and dog health. The development of breast cancer involves multiple signaling pathways, highlighting the need for effective inhibitory drugs that target key proteins in these pathways. This article reviews the dysregulation of the EGFR, PI3K/AKT/mTOR, Hippo, pyroptosis, and PD-1/PD-L1 signaling pathways in HBC and CMT, as well as the corresponding drugs used to inhibit tumor growth, with the aim of providing theoretical support for the development of more efficient drugs.

## 1. Introduction

In 2022, breast cancer surpassed lung cancer as the most prevalent malignant tumor worldwide, with approximately 2.3 million new cases annually, representing 11.7% of all new malignant tumor cases. Among women, breast cancer is the most frequently diagnosed and most lethal of all cancers [1]. The following nine factors have been identified as risk factors for breast cancer: alcohol consumption, body mass index, height, physical activity, mammogram density, age at menarche or menopause, and smoking and type 2 diabetes mellitus (T2DM) [2].

Canines and humans coexist in similar habitats, with comparable biological behaviors and highly analogous genomes. Interestingly, features such as the incidence, hormone levels, tumor size, clinical staging, and lymph node infiltration are also highly analogous in human breast cancer (HBC) and canine mammary tumors (CMT). CMT is the most common type of tumor in dogs. The classification of CMT relies on specific pathological markers, including the presence or absence of significant cellular pleomorphism, necrosis, and lymph node metastasis. Collectively, these markers determine the malignancy grade, which critically influences the health outcomes of affected dogs [3]. Statistically, adult female dogs aged 9–12 years have the highest incidence of CMT, and the causes may include hormonal, nutritional, and genetic factors [4]. Malignant CMT includes predominantly adenocarcinoma, carcinosarcoma, solid carcinoma, papillary carcinoma, or composite carcinoma. CMT can be categorized into four distinct subtypes based on the presence or absence of specific immunohistochemical markers: estrogen receptor (ER), progesterone receptor (PR), and human epidermal growth factor receptor 2 (HER-2 or EGFR-2). These subtypes are as follows: (1) luminal A—characterized by the presence of ER and/or PR, but the absence of HER-2; (2) luminal B—this subtype is marked by the presence of ER and/or PR along with HER-2 positivity; (3) HER-2 enriched—defined by the absence of ER and PR, but the presence of HER-2; (4) triple negative—identified by the lack of all three receptors, ER, PR, and HER-2 [5]. The clinical staging of CMT is determined according to the WHO-approved TNM system, which defines the extension of CMT based on the size of the primary tumor (T), lymph node involvement (N), and the presence of distant metastases (M). One of the most important prognostic factors for CMT is the size of the tumor, which is classified into three groups: T1—less than 3 cm, T2—3-5 cm, and T3—greater than 5 cm. Previously, the biomarkers associated with CMT included Ki-67, PCNA, p53, E-cadherin, CEA, CA15-3, VEGF, EGFR, Her-2, ER, PR, COX-2, *BRCA 1*, *BRCA 2*, and microRNA [6]. The most common treatment is the surgical removal of the CMT [6]. CMT occurs spontaneously in a typical living environment. In addition to the anatomical and physiological similarities between canines and humans, CMT exhibits major pathological features of HBC, including a long-term carcinogenic background, intratumoral heterogeneity, acquired therapy resistance, and distant metastasis [7]. However, compared to humans, canines have a shorter lifespan and more rapid response to cytotoxic drugs. Consequently, the CMT model has become a key tool for the development of innovative anticancer medications (including chemotherapeutic drugs, gene therapy, and biologics), providing invaluable feedback on the efficacy and toxicity of drugs [8] and acting as an exemplary model for human cancer research [9]. Although the lifespan of canines has increased with improved nutrition and the introduction of vaccines, this has also led to a rise in the incidence of canine cancers, with mammary tumors representing the most prevalent type and approximately half of these being malignant.

The development of breast cancer is typically attributed to the dysregulation or mutation of pivotal regulatory proteins, which ultimately leads to the aberrant expression of specific signaling pathways. These signaling pathways are intimately associated with processes such as tumor cell proliferation, angiogenesis, migration, and the remodeling of the microenvironment. A summary of the signaling pathways that may be involved in the regulation of HBC and CMT will provide a theoretical basis for the exploration of breast cancer therapeutic approaches and new ideas for the design and screening of novel therapeutic drugs.

Signaling pathways are frequently studied with a view to identifying key proteins that could serve as potential targets for therapeutic intervention. These proteins, which include kinases and transcription factors, have been shown to play pivotal roles in the proliferation, migration, and invasion of tumor cells. Inhibition of these key proteins can effectively suppress proliferation, angiogenesis, migration, the remodeling of the microenvironment, and immune escape. Thus, the identification of pharmaceutical agents that target these proteins or modulate their aberrant expression would be beneficial in the development of novel anti-breast cancer therapeutics. This review aims to summarize the activation pathways and drugs used in HBC and CMT, clarify the key proteins and their mechanisms of action, and lay a foundation for the subsequent research and development of anticancer drugs.

## 2. Epidermal Growth Factor Receptor (EGFR) Signaling Pathway

### 2.1. Structure and Biological Function of EGFR

The ErbB family of receptors, which includes EGFR (or Her-1), Her-2, Her-3, and Her-4, plays a pivotal role in cell development. However, dysfunctional ErbB can result in aberrant cell proliferation and invasion, which in turn can contribute to the initiation and progression of cancer [10,11,12]. EGFR, a prototypical oncogene, was initially identified in the 1970s [13]. EGFR comprises an extracellular ligand-binding region, a transmembrane region, and an intracellular region. Domains I and III of the extracellular region are responsible for the binding of ligands, including EGF and TGF-α. The formation of an activation complex between EGFR and Her-2 has been shown to enhance signaling pathways [14,15,16].

Upon ligand stimulation, EGFR undergoes dimerization and initiates the phosphorylation of its own tyrosine residues, thereby activating downstream signaling pathways such as the mitogen-activated protein kinase (MAPK), PI3K/AKT/mTOR, and Janus kinase/signal transducer and activator of transcription (JAK/STAT) pathways, which collectively promote cell proliferation and differentiation [17]. However, aberrant activation, gene mutation, or increased ligand expression of the EGFR signaling pathway may result in sustained activation, which is closely associated with invasion, metastasis, and poor prognosis in many malignant tumors [18]. Consequently, EGFR has emerged as a pivotal therapeutic target in cancers such as breast cancer, non-small cell lung cancer, and colorectal cancer [19,20,21].

### 2.2. Mechanisms of Action of EGFR in HBC

The expression and activation of EGFR in breast cancer have been closely associated with specific transcription factors and metabolites. For example, homeobox protein Hox-B5 (HOXB5) has been shown to upregulate EGFR expression and promote the phosphorylation of EGFR and its downstream proteins [22]. Furthermore, recombinant human tubulointerstitial nephritis antigen-like protein 1 (Tinagl1) has been found to bind to EGFR, thereby inhibiting the progression and metastasis of triple negative breast cancer (TNBC), suggesting that Tinagl1 may act as an effective therapeutic agent for TNBC. TNBC is a subtype of breast cancer observed in HBC and CMT that lacks the expression of three key receptors: the estrogen receptor (ER), progesterone receptor (PR), and human epidermal growth factor receptor 2 (HER2). The lack of these receptors makes TNBC particularly challenging to treat with hormone or targeted therapies. TNBC in humans or canines is generally more aggressive, with a higher grade of malignancy and a tendency to metastasize [23]. Protein-tyrosine kinase 7 (PTK7) has been shown to modulate the EGFR/AKT pathway, thereby promoting the proliferation and migration of TNBC cells [24]. In addition, combining anti-EGFR antibodies with neoadjuvant chemotherapy has been shown to remodel the tumor microenvironment by increasing cytotoxic T cells and decreasing immunosuppressive regulatory T cells and M2 macrophages, resulting in higher remission rates [25]. Finally, the plant-derived alkaloid berberine (Berberine) has been shown to impede the proliferation and invasion of TNBC cells by inhibiting the EGFR/MEK/ERK pathway [26].

### 2.3. Mechanisms of EGFR Action in CMT

In the field of CMT research, isorhamnetin, a naturally occurring small-molecule compound derived from plants, has been shown to exert anticancer effects by targeting the EGFR protein and downregulating the EGFR-STST3-PD-L1 pathway in CMT cells [27]. These findings indicate that EGFR can be regulated by specific transcription factors, metabolites, kinases, and plant extracts to inhibit tumor cell proliferation, migration, and invasion by blocking downstream signals, thereby making it an important target for the precise treatment of breast cancer.

### 2.4. Drugs Targeting EGFR

#### 2.4.1. EGFR Tyrosine Kinase Inhibitors (TKIs)

The current generation of EGFR TKIs competitively bind to the ATP-binding sites of active tyrosine kinases, thereby inhibiting the kinase activity of EGFR and blocking downstream signaling pathways. This ultimately leads to the inhibition of TNBC cell proliferation and invasion [28]. The most commonly utilized TKIs include gefitinib (Iressa), erlotinib (Tarceva), lapatinib (Tykerb), and Osimertinib (Tagrisso). Gefitinib, a first-generation selective and reversible TKI, has been shown to induce G1 cell cycle arrest through the inhibition of EGFR kinase activity, which effectively reduces the migration ability of TNBC cells [29]. Moreover, gefitinib has been shown to suppress G-protein-coupled estrogen receptor (GPER) expression, thereby attenuating the stimulatory effect of 17β-estradiol on TNBC growth to a certain extent [30].

Monoinhibitor therapies frequently fail to achieve the desired outcomes, whereas combination therapies have been found to enhance the efficacy. The covalent linkage of tamoxifen and gefitinib has been studied. This combination preserves the ER antagonist activity of tamoxifen. It also maintains the EGFR-inhibitory effect of gefitinib [31]. Furthermore, raloxifene has been shown to potentiate the effects of gefitinib in preventing TNBC growth and metastasis [32]. Gefitinib has been found to be more effective in treating TNBC, whereas erlotinib has been more successful in the treatment of NSCLC. Finally, erlotinib in combination with metformin has been proposed as a potential treatment for metastatic TNBC, although its efficacy in TNBC remains inconclusive [33].

The T790M mutation in EGFR hinders the binding of first-generation TKIs, resulting in the development of resistance [34]. The second-generation TKI lapatinib is a reversible dual inhibitor of EGFR and HER2 that has been shown to overcome some of the resistance. However, the extended administration of lapatinib results in the activation of the PI3K/AKT pathway, ultimately giving rise to secondary resistance, which renders lapatinib an ineffective treatment option for TNBC [35]. Third-generation Osimertinib, the most successful irreversible TKI, has become the treatment of choice for patients with T790M mutations due to its ability to overcome T790M-mediated resistance. However, prolonged treatment with Osimertinib eventually leads to the appearance of the C797S mutation [36].

Fourth-generation TKIs are currently in development, including EGFR variant inhibitors (1,4-aniline quinazoline analogs and amino pyrazolopyrimidine analogs). These novel inhibitors bind to non-catalytic sites on EGFR and can produce synergistic antitumor effects when combined with trastuzumab [37].

#### 2.4.2. Monoclonal Antibodies

**Figure 1:**
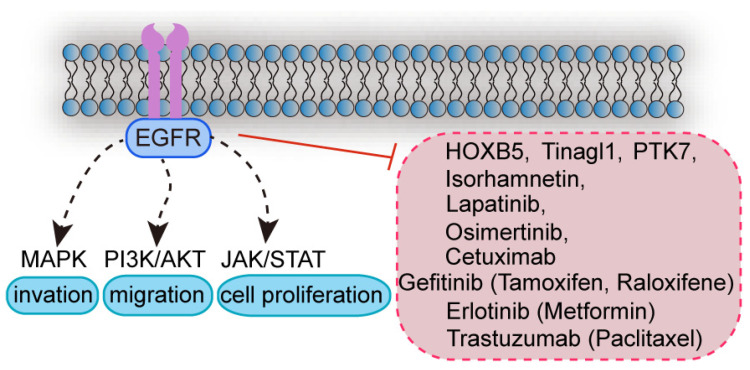
EGFR signaling pathway inhibitors and therapeutic monoclonal antibodies.

Monoclonal antibodies exert their inhibitory effects on tumor cell proliferation by binding to the extracellular ligand-binding region of EGFR, thereby preventing its binding to endogenous ligands and consequently inhibiting the EGFR signaling pathway. Trastuzumab is the first monoclonal antibody to be widely used for the treatment of malignant tumors and has displayed notable efficacy in early-stage breast cancer and the marked prolongation of disease-free survival in patients with TNBC, as evidenced by the analysis of tumor-infiltrating lymphocytes [38]. However, trastuzumab has limited efficacy in advanced breast cancer. Although trastuzumab, when used in combination with paclitaxel, can be effective in treating patients with skin metastases from TNBC, adverse side effects such as the development of brain abscesses have been reported in some patients [39]. Similarly, cetuximab, another targeted therapeutic agent that binds to the extracellular domain of EGFR and inhibits the activation of downstream signaling pathways, has demonstrated efficacy in the treatment of breast cancer by inducing apoptosis in tumor cells. The drug targets associated with the EGFR pathway are summarized in Figure 1.

## 3. The Phosphatidylinositol 3-Kinase/Protein Kinase B/Mammalian Target of Rapamycin (PI3K/AKT/mTOR) Signaling Pathway

The PI3K/AKT/mTOR pathway is a key intracellular signaling pathway that regulates a range of cellular processes, including cell growth, proliferation, apoptosis, angiogenesis, and autophagy. The abnormal activation of this pathway is closely associated with the development of cancer. The PI3K/AKT/mTOR signaling pathway is dysregulated in approximately half of all malignant tumors and has complex relationships with multiple signaling pathways. The abnormal expression of PI3K can also contribute to the development of drug resistance in tumor cells, which significantly impacts the efficacy of therapeutic interventions. Overactivation of the PI3K signaling pathway has been associated with increased breast cancer progression, an increased intratumoral microvessel density, and the enhanced chemotaxis and invasiveness of cancer cells. Consequently, PI3K has emerged as a pivotal target in cancer therapy [40].

The extracellular activation signal of PI3K can be derived from a variety of growth factors and signaling complexes. Fibroblast growth factor (FGF), vascular endothelial growth factor (VEGF), epidermal growth factor (EGF), human growth factor (HGF), vascular locus protein I (Ang1), and insulin all initiate the activation of PI3K.

mTOR kinases form two complexes, mTORC1 and mTORC2, which respond to external stimuli and regulate different downstream targets. mTORC1 comprises mTOR, raptor, deptor, PRAS40, and mLST8/G, mainly in cell growth and energy metabolism. The aberrant activation of mTORC1 can affect the cell status by delaying the G1-S phase of the cell cycle, leading to the occurrence and development of a variety of tumors. mTORC2 is composed of mTOR, Rictor, Sin1, and mLST8, which play a role in cell growth and proliferation and cytoskeletal remodeling, and mTORC2 promotes tumor development by controlling cell proliferation and survival [41].

### 3.1. Inhibitors Targeting PI3K for the Treatment of HBC

Buparlisib (BKM120) is a PI3K inhibitor that has been shown to be efficacious in inducing tumor shrinkage in TNBC. In a phase II clinical trial, 50 patients with metastatic TNBC (mTNBC) exhibited median overall survival (OS) of 11.2 months and median progression-free survival (PFS) of 1.8 months, resulting in a clinical benefit rate of 12% [42]. Capivasertib is another highly selective small-molecule inhibitor targeting AKT1-3 whose efficacy is correlated with the PI3K/AKT activation levels and/or phosphatase and tensin homolog deleted on chromosome 10 (PTEN) status. The preclinical antitumor activity of capivasertib has been validated in animal models, while the efficacy and safety of combining capivasertib with paclitaxel in TNBC patients has been further evaluated in the PAKT trial (NCT03997123) [43].

### 3.2. Inhibitors Targeting AKT for the Treatment of Human HBC

Ipatasertib is a potent small-molecule kinase inhibitor with a high degree of AKT specificity that has demonstrated efficacy in various cancer cells, including ovarian cancer, colorectal cancer (CRC), non-small cell lung cancer (NSCLC), and breast cancer. Its mechanism of action primarily involves ATP competition. Studies have shown that the sensitivity of ipatasertib is predominantly associated with elevated levels of phosphorylated AKT, PIK3CA mutations, and PTEN mutations or deletions. Conversely, KRAS and BRAF mutations have been identified as potential factors contributing to resistance to ipatasertib. Recently, ipatasertib was found to increase the median PFS of patients carrying PIK3CA/AKT/PTEN-mutated tumors to 6.2 months, compared to 3.7 months without ipatasertib (*p* = 0.041), indicating a significant survival benefit [44].

### 3.3. Inhibitors Targeting mTOR for the Treatment of Human HBC

The mTOR inhibitor everolimus has been approved for the treatment of TNBC in combination with iapatinib (NCT01272141), cisplatin (NCT01931163), and carboplatin (NCT02531932) [45]. Combined treatment with everolimus and exemestane was found to be efficacious in patients with hormone receptor-positive, HER2-negative advanced breast cancer, and it extended the median PFS of patients by 4.6 months [46]. In addition, Farmaki et al. reported that using ONC201/TIC10 in combination with everolimus inhibited the growth of drug-resistant cells, thereby providing an effective add-on therapeutic strategy for everolimus-resistant patients with metastatic estrogen receptor-positive (ER+) breast cancer [47].

### 3.4. Drugs That Modulate the PI3K/AKT/mTOR Signaling Pathway in CMT

Homoharringtonine (HHT) is a naturally occurring alkaloid that has been shown to impede the growth of a range of human tumors. However, the inhibitory impact and underlying mechanism of HHT in CMT remain unclear. HHT has been shown to markedly downregulate the expression of p-AKT, p-mTOR, and Bcl-2, while upregulating TP53, Bax, cleaved caspase-3, and caspase-9 expression. Furthermore, HHT has been found to effectively reduce the tumor volume and weight of murine mammary tumors. Together, these studies suggest that HHT may also be effective in inhibiting the PI3K/AKT/mTOR signaling pathway and inducing mitochondrial apoptosis in CMT cells, thereby providing a theoretical basis for clinical treatment [48].

**Figure 2:**
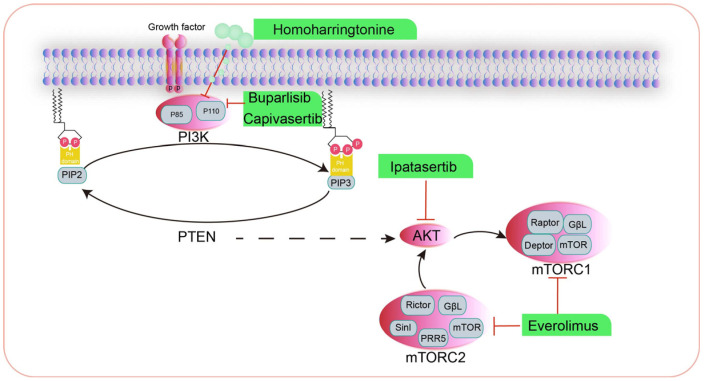
PI3K-AKT-mTOR signaling pathway inhibitors in HBC and CMT.

Recently, Kim et al. conducted whole-exome and transcriptome analyses of 191 spontaneous CMTs with typical HBC features and found close similarities between the HBC and CMT genomes, including a high frequency of PIK3CA mutations (43.1%) and aberrations in the PI3K/AKT pathway (61.7%). Three distinct gene expression-based CMT subtypes were identified, one of which exhibited a high degree of similarity to the human basal-like breast cancer subtype. This subtype is characterized by active epithelial–mesenchymal transition (EMT), low-density lipoprotein expression, and notable differences in disease prognosis. However, the relative paucity of ERBB2 amplification and Her2-enriched isoforms in CMT is indicative of species-specific molecular mechanisms. The findings of this study offer novel insights into the oncogenic characteristics of cross-species breast cancer and establish a foundation for precise diagnosis and treatment in canines [49]. Tae-Min’s study found striking similarities in genomic signatures by performing a whole-exome and transcriptome analysis of 191 spontaneous CMT that exhibited archetypal features of HBC, including frequent PIK 3CA mutations (43.1%), aberrations in the PI3K-Akt pathway (61.7%), and key genes involved in cancer initiation and progression. Three subtypes of CMT were also identified, one of which was similar to the basal-like HBC subtype, with activated epithelial–mesenchymal transition, low claudin expression, and an unfavorable disease prognosis [50]. Drug targets associated with the PI3K/AKT/mTOR pathway are summarized in Figure 2.

## 4. Cell Death Signaling Pathway

Cellular pyroptosis is a form of programmed cell death that is accompanied by an inflammatory response that promotes the release of immunogenic substances and attracts immune cells, thereby transforming “cold” tumors into “hot” tumors. The first studies demonstrating that Shigella fumigatus and Salmonella typhimurium could trigger the lysogenic death of macrophages were conducted in the 1990s [50,51]. Subsequent studies demonstrated that this type of cell death was dependent on caspase-1 activation and was distinct from the caspase-3-dependent apoptosis observed in traditional apoptosis [52]. Cellular pyroptosis is mediated by the gasdermin (GSDM) family. Recent studies have shown that pyroptosis plays a pivotal role in tumor progression and may represent a novel strategy for cancer treatment.

### 4.1. Classical Tumor Cell Pyroptosis Pathways

The classical cellular pyroptosis pathway is largely dependent on the NLRP3 inflammasome and is regulated through GSDMD. Caspase-1 activates GSDMD by removing its C-terminal inhibitory fragment. Another crucial aspect of GSDMD-dependent pyroptosis is the release of IL-1β and IL-18 via non-classical secretory pathways [53]. Cisplatin has been shown to induce pyroptosis in TNBC cells via the MEG3/NLRP3/caspase-1/GSDMD pathway [54]. In addition, Xing et al. have developed an extracellular vesicle-based GSDMD-N mRNA delivery system, which effectively enhanced the effectiveness of cancer immunotherapy [55]. In CMT, only Zhu reported that resveratrol activates NK cells and enhances the antitumor effect by activating NLRP 3/caspase-1/cytochrome C/GSDMD pathway [56].

### 4.2. Non-Classical Pathways That Regulate Inflammatory Cell Death

In addition to the classical pathway, inflammatory cell death can also be induced by the non-classical pathway, which is mainly mediated by the activation of caspase-4, caspase-5, or caspase-11 by LPS. The non-classical pathway can also activate the NLRP3/caspase-1 pathway, which promotes the maturation and secretion of IL-1β and IL-18 [57]. Moreover, N-GSDMD cleavage by caspase-4/-5/-11 results in a reduction in the intracellular potassium levels, which subsequently activates the NLRP3 inflammasome [58]. Quercetin has been shown to induce pyroptosis in MDA-MB-231 cells following photodynamic and photothermal modifications [59].

### 4.3. Other Pathways

Certain pharmaceutical agents can stimulate tumor cells to undergo pyroptosis via the caspase-3/GSDME pathway. For example, Haein et al. showed that tetra-arsenohexoxide induces the caspase-3/GSDME pathway through the activation of mitochondrial reactive oxygen species, resulting in the inhibition of tumor growth and metastasis in TNBC cells [60]. Moreover, Yang et al. reported that histone deacetylase inhibitors could activate the caspase-3/GSDME pathway to induce cellular pyroptosis by interfering with glutathione metabolism, resulting in cell cycle arrest and alterations in the intracellular redox balance [61].

### 4.4. Drugs That Induce Cellular Pyroptosis in HBC

To date, a range of small-molecule compounds, drugs, and nanomedicines have been identified that can induce pyroptosis in breast cancer cells and thus provide a novel approach for the treatment of breast cancer. The specific details of these drugs can be found in Table 1.

### 4.5. Drugs That Induce Cellular Apoptosis in CMT

At present, CMT is more preferably treated with surgical resection. Andressa used feroxib as a neoadjuvant treatment for CMT and then surgically removed the canine mammary gland on the 10th day after treatment, and the results showed that feroxib could increase the number of COX-2-positive cells undergoing apoptosis and inhibited CMT growth [68]. Madalina’s study observed the effects of adriamycin on two CMT cell lines, P114 and CMT-U27. The result indicated that adriamycin showed an inhibitory effect on cell proliferation and altered the expression of EMT-related genes in both cell lines [69]. Fikriye demonstrated the clinical efficacy and reliability of two different neoadjuvant chemotherapy (NAC) regimens consisting of doxorubicin/cyclophosphamide (AC) and paclitaxel in dogs with clinical stage II-IV CMT in their study. The results showed that the complete response (CR) to TNBC in the AC group was 33%, whereas no CR was observed with paclitaxel [70].

## 5. Immunotherapy with Programmed Death-1/Programmed Death Ligand-1 (PD-1/PD-L1) as Immune Checkpoints

The PD-1 protein plays a significant role in the growth of cancer cells. PD-1 was first discovered in 1992 by Tasuku Honjo and colleagues, who reported that PD-1 was expressed in activated T cells, natural killer cells, and B cells [71]. PD-1 has since been shown to interact with PD-L1, which is expressed in a range of tumor cells, as well as playing a significant role in suppressing the immune system in mice [72]. Studies by Chen et al. have shown that the interaction between PD-L1 and PD-1 inhibits T cell activity, preventing them from attacking cancer cells [73].

PD-1/PD-L1 inhibitors have been used in the treatment of advanced breast cancer [74,75]. The small-molecule immunosuppressant BMS-202 was found to markedly enhance the efficacy of immunotherapy by inhibiting the binding of PD-1 to PD-L1 [76]. Zeng et al. developed a nanoplatform integrating photodynamic therapy and BMS-202, which exhibited remarkable therapeutic efficacy against tumors and their metastases [77]. Moreover, monoclonal antibodies such as atezolizumab and pembrolizumab have demonstrated efficacy against both early-stage and advanced/metastatic TNBC [78]. While one study indicated that atezolizumab in conjunction with paclitaxel did not enhance PFS and OS in individuals with TNBC [79,80], another study demonstrated that its combination with neoadjuvant chemotherapy markedly elevated the rate of pathological complete remission [81]. At present, there is no monoclonal antibody targeting PD-1/PD-L1 currently available for use in the treatment of CMT. However, research in this area is ongoing. Drugs associated with these pathways are summarized in Table 2.

## 6. Antibody–Drug Conjugates (ADCs)

ADCs are a novel class of antitumor agents that employ the antibody-mediated delivery of highly potent cytotoxic drugs to tumor cells, thereby reducing the toxicity associated with conventional chemotherapeutic agents. A number of HER2-ADC products have been granted approval, with Kadcyla (trastuzumab emtansine) and Enhertu (trastuzumab deruxtecan) approved for the treatment of HER2-positive breast cancer.

Kadcyla, a combination of a humanized anti-HER2 antibody with the microtubule inhibitor DM1, has been primarily used for the adjuvant treatment of HER2-positive early-stage breast cancer. In contrast, Enhertu has been approved for the treatment of patients with metastatic HER2-positive breast cancer who have previously undergone treatment with anti-HER2 drugs. This is achieved through the coupling of an anti-HER2 antibody with a topoisomerase I inhibitor [82]. In addition, researchers have developed an ADC of trastuzumab coupled to the microtubule-disrupting agent MMAE (trastuzumab-MC-Val-Cit-PABC-MMAE), which has been shown to exhibit high specificity and antitumor activity against HER2-positive tumor cells [83].

**Figure 3:**
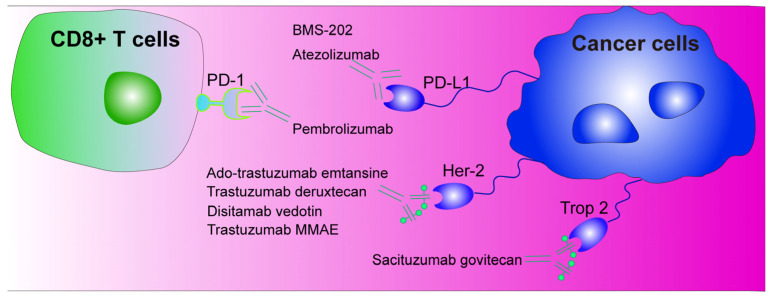
Antibody–drug conjugates for the treatment of mammary cancer.

Trop-2 has also been identified as an emerging target in breast cancer. The Trop-2 antibody-coupled drug sastuzumab (Trodelvy) has been approved for use in HR+/HER2-advanced breast cancer patients [84]. Studies have shown that sacituzumab govitecan could markedly extend the PFS and OS in patients with metastatic TNBC [85]. Furthermore, Fan et al. reported that disitamab vedotin (RC48), an ADC drug that targets Her2, demonstrated promising efficacy in both Her2-positive and low-expression breast cancer [86,87]. The future development of ADC drugs will focus on improving their specificity, enhancing their efficacy, and reducing their toxicity. Future research can be carried out through target optimization; the development of antibodies with dual or multi-target recognition capabilities; the adjustment of drug loading; the optimization of the toxin properties to make it easier to accumulate in cells and reduce the likelihood of efflux; the combination of ADCs with immune checkpoint inhibitors, cytokine therapy, or traditional chemotherapy; and the selection of the most suitable ADC target by analyzing the gene expression profile and phenotypic data of cancer patients. At present, there is a paucity of evidence regarding the use of immunotherapeutic drugs in the context of CMT. However, it is anticipated that future advances in this field will contribute to the enhancement of clinical management strategies for pets. Drug targets associated with the ADCs pathway are summarized in Figure 3.

## 7. Conclusions

CMT and HBC have similarities in their disease characteristics, subtype distribution, and treatment strategies, especially in the study of TNBC and hormone-driven breast cancer. As with HBC, different subtypes, such as ER/PR-positive, HER2-positive, and TNBC, have been observed in CMT. Both CMT and HBC occur in complex microenvironments that influence the growth and metastasis of cancer cells. The immune response in CMT is very similar to that in HBC, especially in terms of the immune infiltration and immune escape mechanisms. CMT exhibits abnormalities in many HBC-related genes, such as HER2 overexpression and PI3K/AKT/mTOR signaling pathway activation. CMT is also strongly associated with hormones, especially in unneutered female dogs. Hormone levels directly affect the incidence of CMT.

The difference between CMT and HBC is that the incidence of CMT is significantly higher in unneutered female dogs than in human females, and the prevalence is reduced substantially after neutering. This is closely related to the reproductive physiology and hormone levels of dogs. Canine TNBC is highly similar to human TNBC in terms of the immune microenvironment, aggressiveness, and metastasis pattern, and it can be used to study the mechanisms of tumor invasion and immunotherapy. The canine TNBC model can help to evaluate the effectiveness of novel treatments such as immunotherapy and chemotherapy combination therapy, providing a realistic and reliable model for translational research in breast cancer. Studying the characteristics of CMT in depth can provide important insights and support for HBC research, especially for aggressive cancers and the development of novel treatments.

Despite the targeting of a range of signaling pathways and transcription factors in breast cancer therapy, the treatment of breast cancer is constantly confronted with rapidly evolving challenges, including the emergence of drug-resistant cell lines. The efficacy of therapeutic interventions is constrained by differences in the specificity of each treatment modality and the population to which it is adapted. Future studies can optimize drug combinations in conjunction with clinical needs to fully utilize the anticancer potential of drugs and improve their therapeutic efficacy. As one of the most common malignancies in female dogs, no drugs have been developed specifically for the treatment of CMT. To date, there are no reports in the literature on the direct use of HBC drugs in dogs. This may be due to genomic differences between canines and humans, which result in variations in therapeutic targets. Nevertheless, this is a very promising idea that could lead to laboratory research and accelerate the development of therapeutic drugs for CMT.

Currently, personalized medicine is gaining significant attention. The main goal of personalized medicine is to select the correct dose of the right drug at the right time according to the specific needs of the patient. Currently, modern personalized medicine is moving towards targeted therapy. The prerequisite for targeted treatment is to determine whether the protein of interest is highly expressed in the individual and thus to identify the targeted therapeutic agent. These targeted proteins play crucial roles in the signaling pathways that drive tumor progression, underscoring the need for further research to uncover novel targets and improve the treatment precision.

## Figures and Tables

**Table 1:** Drugs targeting cellular pyroptosis in breast cancer.

Pyroptosis Inducer	Cell Line	Pyroptosis Pathway	Year
Cadmium [62]	MDAMB231	Caspase-3/GSDME	2021
Dihydroartemisinin [63]	MCF7, MDAMB231	Caspase-3/GSDME	2021
Cinobufagin [64]	MDA-MB-231, 4T1	Caspase-3/GSDME	2023
Fe-ZnO2@HA [65]	4T1	Caspase-1/GSDMD	2024
PARPi [66]	MDAMB436, HCC1937	Caspase-8/GSDMC	2024
L@NBMZ [67]	MCF-7	Caspase-3/GSDME	2024

The numbers in [] are the corresponding reference numbers.

**Table 2:** Drugs used in different pathways to kill cancer cells.

Drug	Pathway	Cell Species	Outcome
Gefitinib [29]	EGFR tyrosine kinase inhibitors	TNBC	G1 cell cycle arrest
Raloxifene [32]	EGFR tyrosine kinase inhibitors	TNBC	Cell apoptosis
Lapatinib [35]	EGFR tyrosine kinase inhibitors	(EGFR+, HER-2+) HBC	Downregulation of CDK6 and DNMT1
Trastuzumab [37]	EGFR monoclonal antibodies	HER2+ HBC	Cell apoptosis
Buparlisib [42]	PI3K inhibitor	TNBC	Cell apoptosis
Capivasertib [43]	AKT1-3 inhibitor	TNBC	Cell cycle arrest
Ipatasertib [44]	AKT inhibitor	TNBC	Cell cycle arrest
Homoharringtonine [48]	PI3K/AKT/mTOR inhibitor	CMT	Cell apoptosis
BMS-202 [76]	PD-1/PD-L1 inhibitor	TNBC	Cell apoptosis
Atezolizumab [79,80]	PD-L1 inhibitor	TNBC	Cell autophagy

The numbers in [] are the corresponding reference numbers.

## Data Availability

Not applicable.
